# Supplementary material for: IFITM proteins promote SARS-CoV-2 infection and are targets for virus inhibition in vitro
Source: Nat Commun. 2021 Jul 28;12:4584. doi: 10.1038/s41467-021-24817-y (PMC8319209; doi:10.1038/s41467-021-24817-y)
Supplement: Supplementary file 1 — Supplementary Information [file 41467_2021_24817_MOESM1_ESM.pdf]

Supplementary information for

IFITM proteins promote SARS-CoV-2 infection and are targets for virus inhibition in vitro

Caterina Prelli Bozzo[1#], Rayhane Nchioua[1#], Meta Volcic[1], Lennart Koepke[1], Jana Krüger[2], Desiree Schütz[1], Sandra Heller[2], Christina M. Stürzel[1], Dorota Kmiec[1,3], Carina Conzelmann[1], Janis Müller[1], Fabian Zech[1], Elisabeth Braun[1], Rüdiger Groß[1], Lukas Wettstein[1], Tatjana Weil[1], Johanna Weiß[1], Federica Diofano[4], Armando A. Rodríguez Alfonso[5,6], Sebastian Wiese[6], Daniel Sauter[1,7], Jan Münch[1], Christine Goffinet[8], Alberto Catanese[9], Michael Schön[9], Tobias M. Boeckers[9,10], Steffen Stenger[11], Kei Sato[12], Steffen Just[4], Alexander Kleger[2], Konstantin M.J. Sparrer[1*] and Frank Kirchhoff[1*]

[1]Institute of Molecular Virology, Ulm University Medical Center, 89081 Ulm, Germany. [2]Department of Internal Medicine I, Ulm University Medical Center, 89081 Ulm, Germany. [3]Department of Infectious Diseases, King's College London, WC2R 2LS London, United Kingdom. [4]Department of Internal Medicine II (Cardiology), Ulm University, 89081 Ulm, Germany. [5]Core Facility of Functional Peptidomics, Ulm University Medical Center, 89081 Ulm, Germany. [6]Core Unit of Mass Spectrometry and Proteomics, Ulm University Medical Center, 89081 Ulm, Germany. [7]Institute of Medical Virology and Epidemiology of Viral Diseases, University Hospital Tübingen, 72076 Tübingen, Germany. [8]Institute of Virology, Charité - Universitätsmedizin Berlin, 10117 Berlin, Germany. [9]Institute for Anatomy and Cell Biology, Ulm University, 89081 Ulm, Germany. [10]Deutsches Zentrum für Neurodegenerative Erkrankungen (DZNE), Ulm University, 89081 Ulm, Germany. [11]Institute of Medical Microbiology and Hygiene, Ulm University Medical Center, 89081 Ulm, Germany. [12]Institute of Medical Science, The University of Tokyo, 1088639 Tokyo, Japan.

# contributed equally

* Correspondence: Konstantin.Sparrer@uni-ulm.de or Frank.Kirchhoff@uni-ulm.de

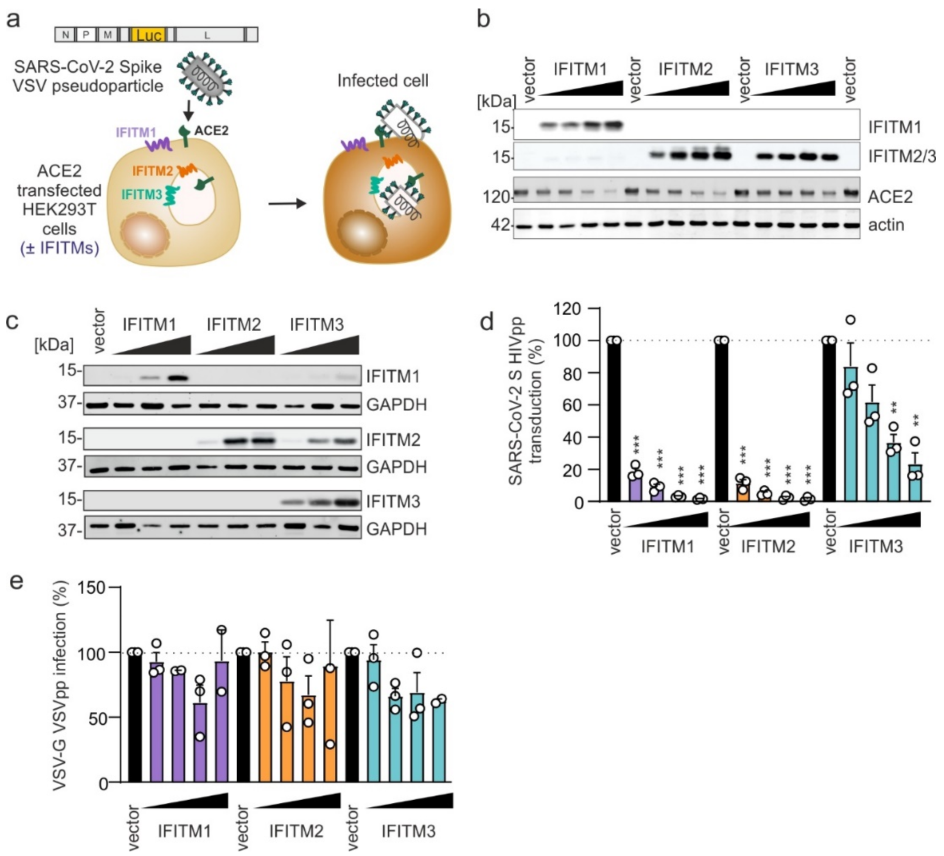

Supplementary Figure 1. Effect of IFITM overexpression in the target cells on Spike- or VSV-G-mediated pseudoparticle (VSVpp) infection. a, Schematic depiction of the assay to assess VSVpp entry. **b**, Exemplary immunoblot of whole cell lysates (WCLs) of HEK293T cells co-transfected with expression plasmids for ACE2 or different doses of IFITM expression constructs. Blots were stained with anti-ACE2, anti-IFITM1, anti-IFITM2/3 and anti-actin. The experiment was repeated thrice with similar results. **c,** Expression of IFITM1, IFITM2 and IFITM3 in HEK293T cells transiently transfected with serial dilutions of the expression vectors. Exemplary immunoblot of whole cell lysates (WCLs) of transiently transfected HEK293T cells, as indicated. The experiment was repeated twice with similar results. **d**, Quantification of SARS-CoV-2-S-mediated entry by measuring luciferase activity in HEK293T cells transiently transfected with the indicated expression vectors and transduced 24 h post-transfection with HIV(luc)Δenv*-SARS-CoV-2 S for 48 h. **e**, VSV(luc)ΔG*VSV-G entry in HEK293T cells transiently expressing the indicated proteins and infected 24 h post-transfection with VSV(luc)ΔG*VSV-G (MOI 0.025) for 16 h. All bar diagrams in this figure represent means of three independent experiments (±SEM), unpaired t test with Welch´s correction, exact p values are provided in the Supplementary Data 1. P values are indicated as *, $p < 0.05$; **, $p < 0.01$; ***, $p < 0.001$; or were not significant ($p > 0.05$). Related to Fig. 1.

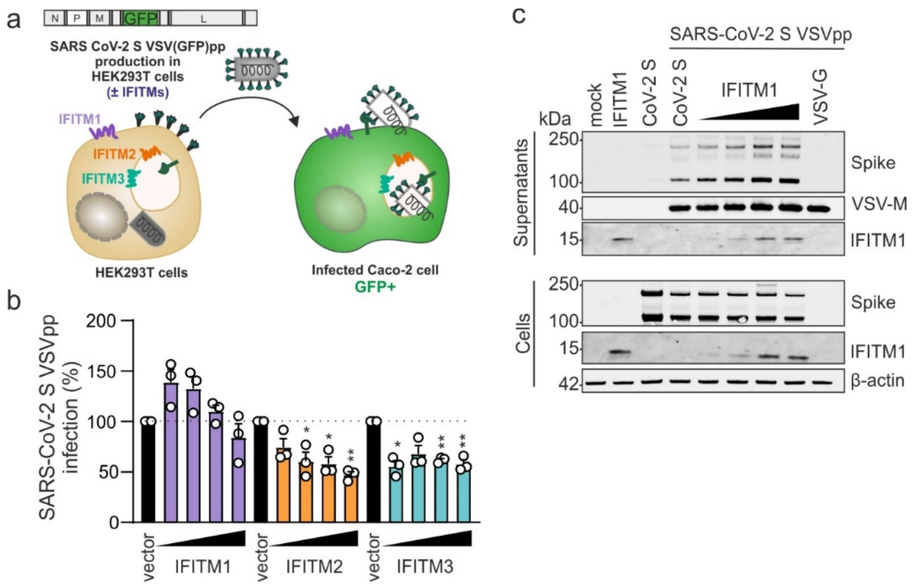

Supplementary Figure 2. Effect of IFITM overexpression in the producer cells on Spike-mediated VSV pseudoparticle infection. a, Schematic depiction of the assay to assess VSVpp production and infection. **b,** Quantification of SARS-CoV-2-S-mediated entry by measuring luciferase activity in Caco-2 cells transduced 24 h post-transfection with VSV(luc) ΔG*SARS-CoV-2- S (MOI 0.025) for 16 h. Bars represent the mean of three independent experiments (±SEM), unpaired t test with Welch´s correction, exact p values are provided in Supplementary Data 1. **c**, Immunoblot of supernatants and whole cell lysates (WCLs) of HEK293T cells co-transfected with expression plasmids for SARS-CoV-2 Spike and increasing doses of IFITM1 expression construct. The experiment was performed once. P values are indicated as *, p < 0.05; **, p < 0.01; ***, p < 0.001; or were not significant (p > 0.05). Related to Fig. 1.

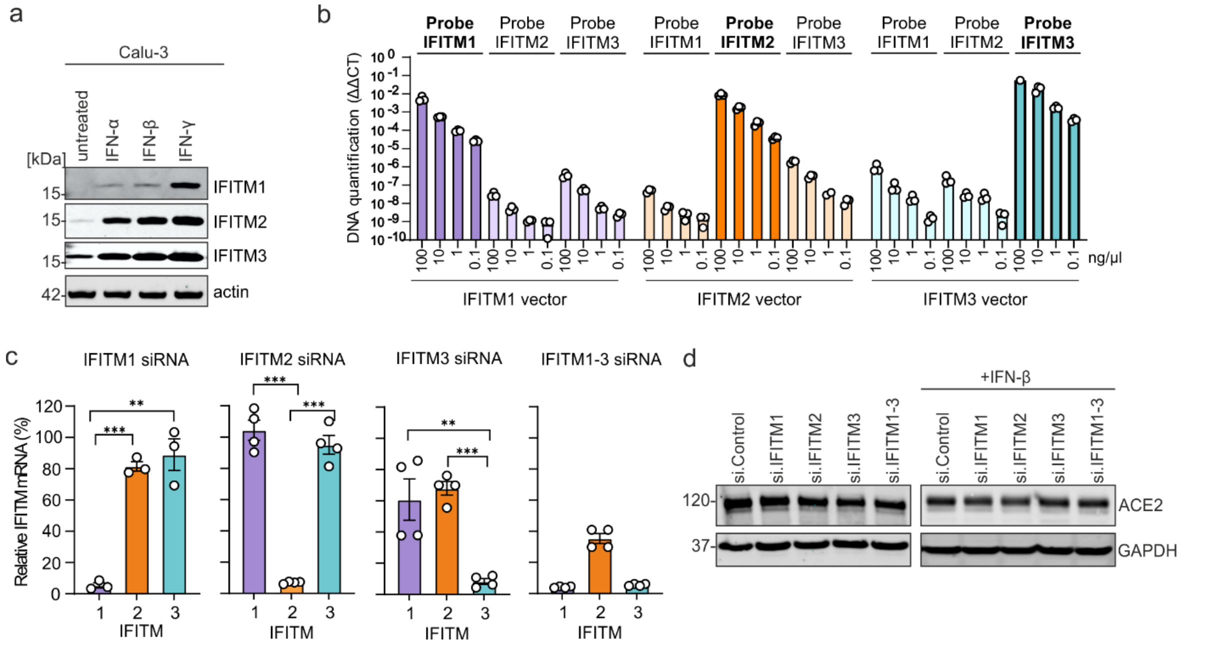

Supplementary Figure 3. IFITM expression, siRNA knock-down and antibody specificity. a, Expression of IFITM1, IFITM2 and IFITM3 in Calu-3 cells after stimulation with IFN-α (500 U/ml, 72 h), IFN-β (500 U/ml, 72 h) or IFN-γ (200 U/ml, 72 h). Immunoblots of whole cell lysates were incubated with anti-IFITM1, anti-IFITM2, anti-IFITM3 and anti-actin. The experiment was performed once. **b,** Relative quantification of the indicated serial dilutions of IFITM1, 2 and 3 expression vectors via qRT-PCR using specific primer/probe sets as indicated. Bars represent the mean of three independent experiments (±SEM). **c,** IFITM mRNA quantification in Calu-3 cells treated with control (CTRL) or IFITM1, 2 and/or 3 targeting siRNA treated with IFN-β. Bars represent the mean of three (IFITM1 siRNA panel) or four (IFITM2 and IFITM3 siRNA panels) independent experiments each measured in technical duplicates (±SEM), unpaired t test with Welch´s correction, exact p values are provided in the Supplementary Data 1. **d,** Immunoblot analysis of ACE2 expression in Calu-3 cells left unstimulated (left panel) or stimulated with IFN-β (right panel) and transfected with control (CTRL) or IFITM1, 2 and/or 3 targeting siRNAs. The experiment was performed two times independently. P values are indicated as *, $p < 0.05$; **, $p < 0.01$; ***, $p < 0.001$; or were not significant ($p > 0.05$). Related to Fig. 1.

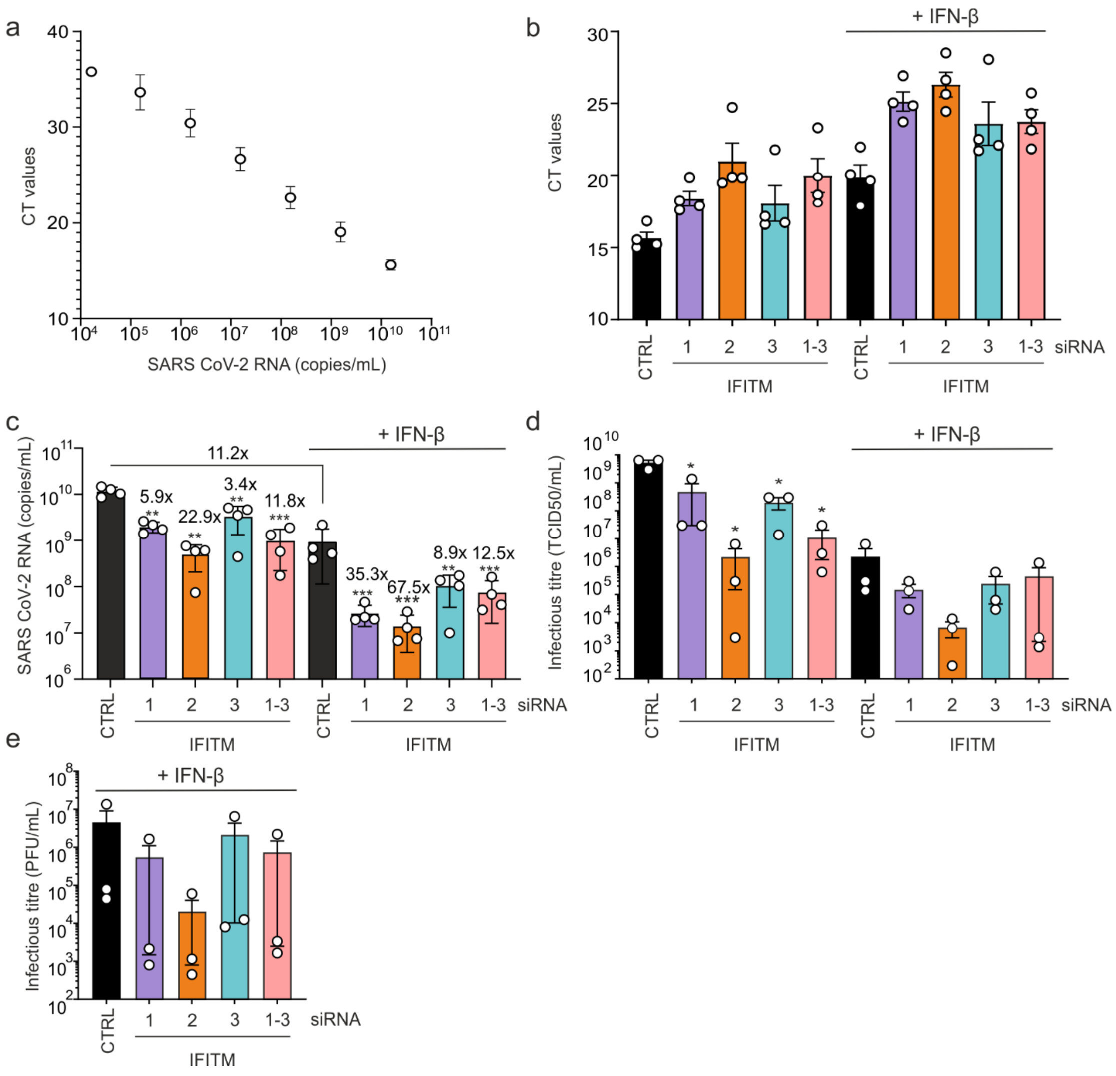

Supplementary Figure 4. Impact of IFITM siRNA knock-down on SARS-CoV-2 replication in Calu-3 cells. a-c, Standard curve (a), raw qRT-PCR CT values (b) and SARS-CoV-2 RNA copy numbers (c) in the supernatant of Calu-3 cells collected 2 days post-infection with SARS-CoV-2 (MOI 0.05). Relative levels of viral RNA production are shown in Fig. 1d. Number above the bars indicate n-fold reduction of viral RNA levels upon knockdown of the respective IFITM proteins compared to cells treated with control siRNA or fold inhibit by IFN-β treatment, respectively. Bars represent mean values (±SEM) of four independent experiments each measured in technical duplicates. **d**, TCID50/mL values of Fig. 1e. Bars represent mean values (±SEM) of three independent experiments. **e**, plaque forming units (PFU)/mL values of Fig. 1g. Bars represent mean values (±SEM) of three independent experiments. (c-d) Paired t-test, exact p values are provided in Supplementary Data 1. P values are indicated as *, $p < 0.05$; **, $p < 0.01$; ***, $p < 0.001$; or were not significant ($p > 0.05$). Related to Fig. 1.

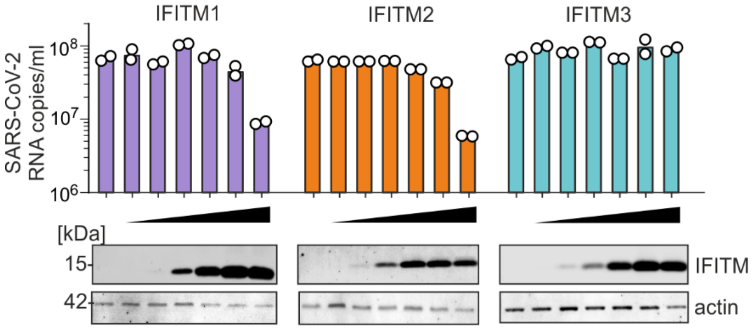

Supplementary Figure 5. Effect of different levels of transient IFITM expression on SARS-CoV-2 infection. SARS-CoV-2 RNA production from HEK239T cells transiently expressing ACE2 and increasing levels of the indicated IFITM proteins. Quantification of viral N gene RNA by qRT-PCR in the supernatant of HEK293T was performed 48 h post-infection with SARS-CoV-2 (MOI 0.05). Bars represent means of two independent experiments. Related to Fig. 1.

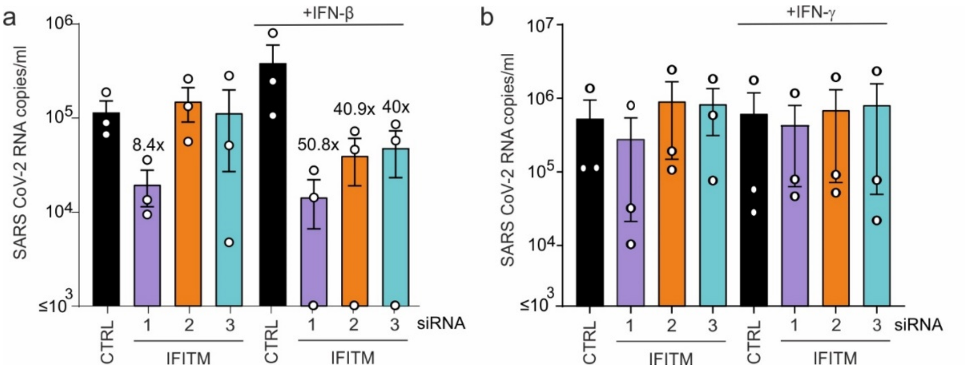

Supplementary Figure 6. Impact of IFITM siRNA knock-down on SARS-CoV-2 replication in SAEC cells. a, **b**, SARS-CoV-2 RNA copy numbers in the supernatant of SAEC cells that were left untreated or treated with (a) IFN-β or (b) IFN-γ collected 2 days post-infection with SARS-CoV-2 (MOI 2.5). Relative viral RNA levels are shown in Figs. 2b and 2c. Bars represent mean values of three independent experiments each measured in technical duplicates (±SEM). Related to Fig. 2.

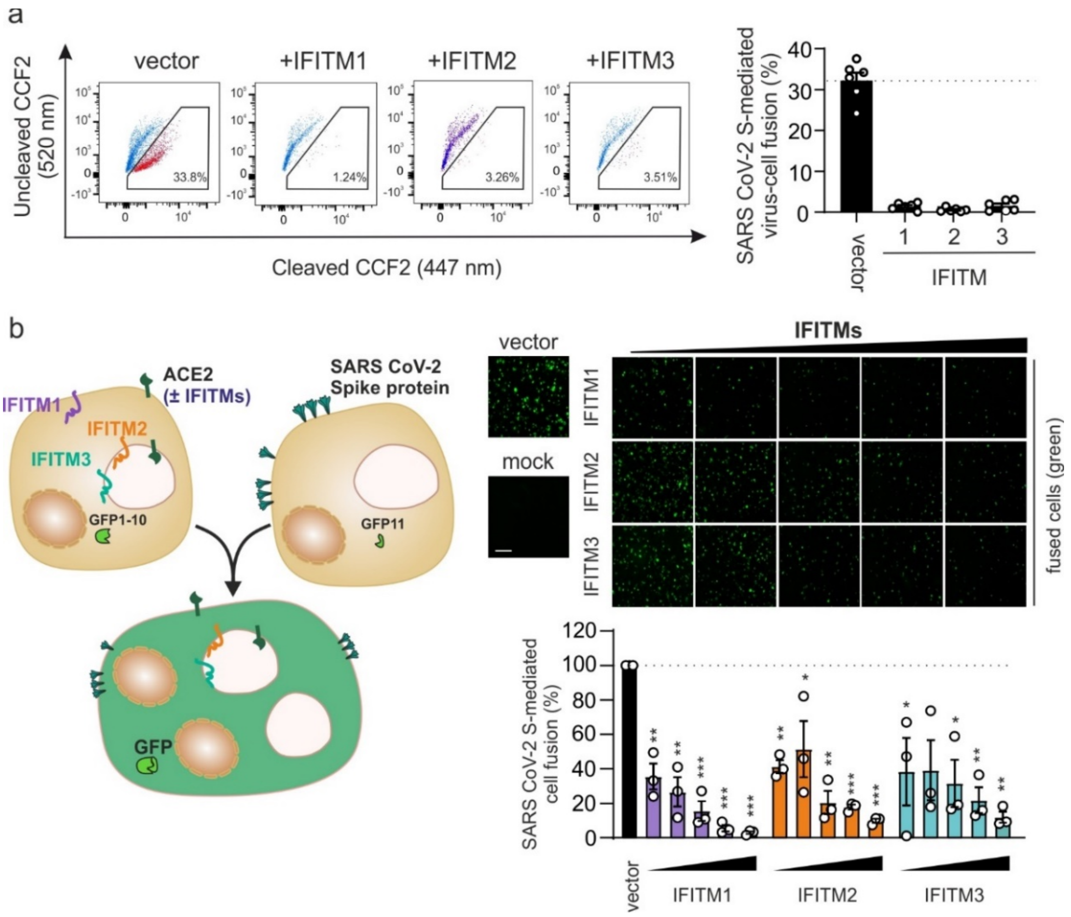

Supplementary Figure 7. Overexpression of IFITMs prevents S-mediated virion and cell-to-cell fusion. a, Fusion of HIV(Vpr-Blam)Δenv*-SARS-CoV-2-S with HEK293T cells transiently expressing ACE2 and IFITMs. Quantification of the fusion efficiency by flow cytometry as percentage of (cleaved CCF2) positive cells. Bars represent means (±SEM) of three independent experiments each done in technical duplicates. Left panel shows exemplary gating of the raw data. **b**, Schematic outline of the split-GFP assay measuring cell-cell fusion (left panel). GFP1-11 and SARS-CoV-2 Spike expressing HEK293T were co-cultured with GFP10, ACE2 and IFITM expressing HEK293T. Exemplary fluorescence images (upper). Quantification of successful fusion by GFP positive cells (green) normalized to nuclei (lower right). Bars represent means of three independent experiments (±SEM). Unpaired t test with Welch´s correction, exact p values are provided in Supplementary Data 1. Scale bar, 20 µm. P values are indicated as *, $p < 0.05$; **, $p < 0.01$; ***, $p < 0.001$; or were not significant ($p > 0.05$). Related to Fig. 2.

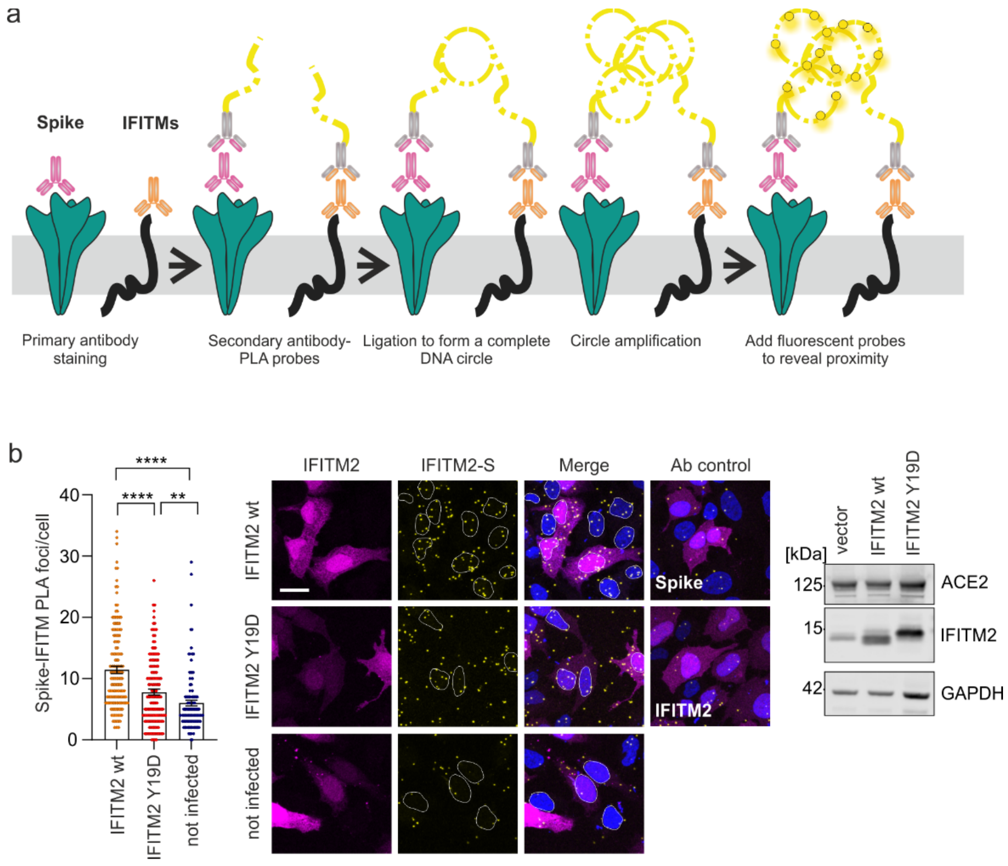

Supplementary Figure 8. Schematic outline of the Proximity Ligation Assay (PLA) and impact of a Y19D mutation in IFITM2 on proximity to the Spike protein. a, Schematic representation of the PLA assay used to analyze the proximity between SARS-CoV-2 Spike and IFITM proteins. **b,** Proximity ligation assay between the SARS-CoV-2 Spike and IFITM2 protein in Hela-ACE2 cells infected with SARS-CoV-2 for 2 h at 4°C. Nuclei, DAPI (blue). Cells were stained for IFITM2 (magenta), PLA signal (yellow) for Spike/IFITM2. Scale bar, 20 µm. (middle panel) Quantification of the PLA signal. Bars represent the mean, dots individual counted cells (±SEM) over four independent experiments, exact p values are provided in Supplementary Data 1. Bars represent the mean of four representative experiments using four individual images (200 cells ±SEM), two sided Wilcoxon matched-pairs test, exact p values are provided in Supplementary Data 1. (left panel) Exemplary immunoblot showing ACE2 and IFITM2 expression in Hela-ACE2 cells transfected with wild-type and Y19D mutant IFITM2 expression constructs or an empty control vector. The signal in the vector lane in the right panel results from endogenous IFITM2 expression. Related to Fig. 4.

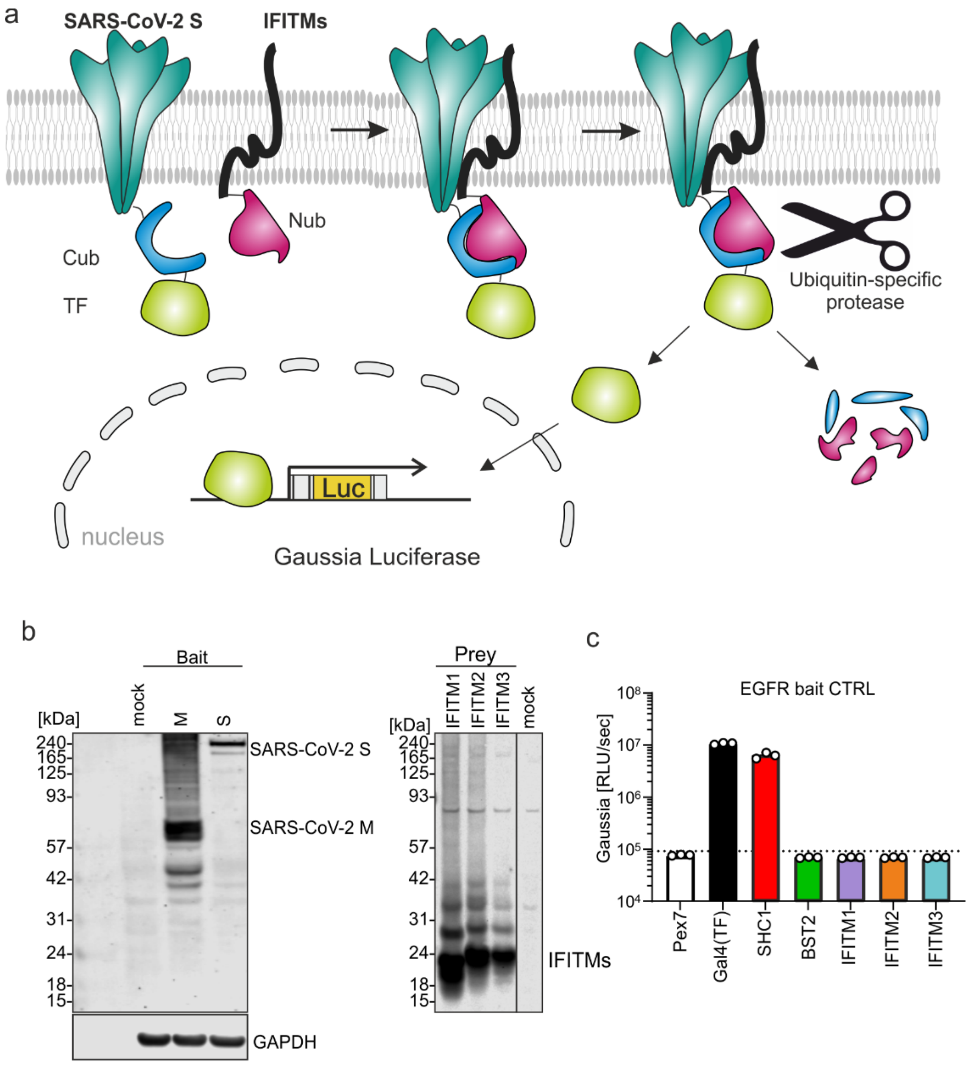

Supplementary Figure 9. Analysis of protein-protein interactions by MaMTH assay. a, Schematic representation of the MaMTH assay measuring interactions between SARS-CoV-2 Spike and IFITM1, 2 or 3.Cub: C-terminal half of ubiquitin, Nub: N-terminal half of ubiquitin, TF: Transcription factor, Luc: luciferase. **b,** Exemplary immunoblot showing the expression of MaMTH V5-tagged SARS-CoV-2 protein baits and FLAG-tagged IFITM preys in transfected HEK293T B0166 cells. Whole cells lysates were stained for SARS-CoV-2 S and M as well as IFITMs (FLAG) and GAPDH. Western blots were performed once. **c**, Raw values of negative (Baits only or Preys with EGFR Bait) and positive controls (transcription factor Gal4 or EGFR with SHC1) used in the MaMTH protein-protein interaction assay. The dotted line indicates values detected in mock transfected cells. Shown are mean values of two independent experiments each measured in triplicates. Related to Fig. 4.

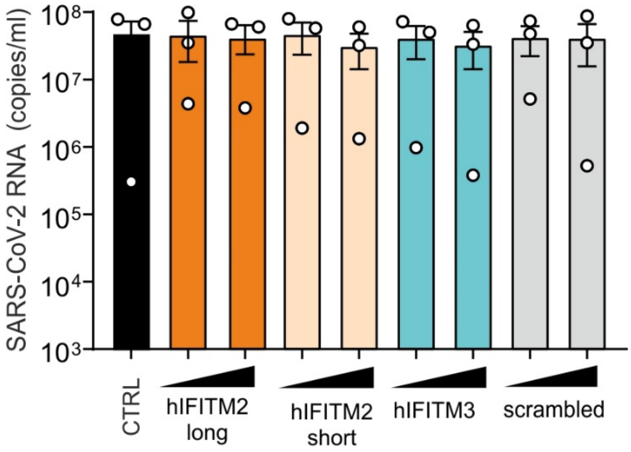

Supplementary Figure 10. Effect of IFITM-derived peptides on SARS-CoV-2 infectivity. Quantification of Viral N gene RNA levels in the supernatant of Calu-3 cells infected with SARS-CoV-2 pre-treated with two concentrations of IFITM-derived peptides by qPCR. Bars represent the mean of three independent experiments measured in technical duplicates (±SEM). The experiment was repeated independently three times with similar results. Related to Fig. 5.

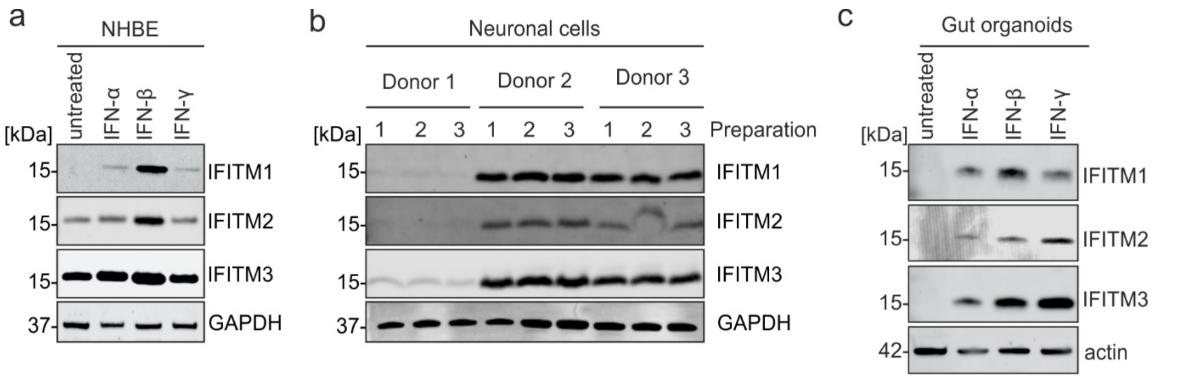

Supplementary Figure 11. Expression of IFITMs in primary lung cells, neuronal cells and gut organoids. a, Exemplary immunoblots showing expression of IFITM1, IFITM2 and IFITM3 in primary bronchial epithelial cells (NHBE) after stimulation with IFN-α (500 U/ml, 72 h), IFN-β (500 U/ml, 72 h) or IFN-γ (200 U/ml, 72 h). Blots were stained with anti-IFITM1, anti-IFITM2, anti-IFITM3 and anti-GAPDH. **b,** Expression of IFITM1, IFITM2 and IFITM3 in neuronal cells analyzed as in (a). Cell lysates were prepared from three independent differentiations of induced pluripotent stem cells of three different donors. **c**, Expression of IFITM1, IFITM2 and IFITM3 after stimulation with IFN-α (500 U/ml, 72 h), IFN-β (500 U/ml, 72 h) or IFN-γ (200 U/ml, 72 h) in stem cell derived gut organoids. (a-c) All Western blots were performed once. Related to Fig. 6.

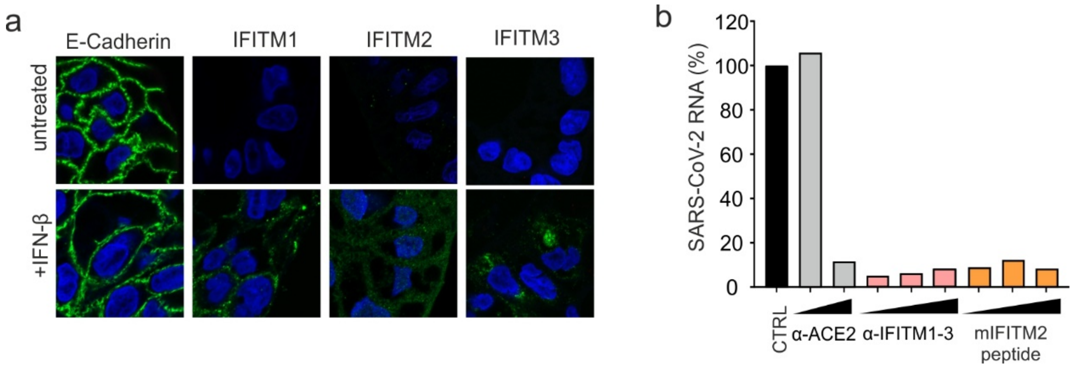

Supplementary Figure 12. Inhibition of SARS-CoV-2 infection in gut organoids a, Representative immunofluorescence images of stem cell-derived gut organoids before and after stimulation with IFN-β (500 U/ml, 72 h). Cells were stained with anti-E-Cadherin, anti-IFITM1, anti-IFITM2 or anti-IFITM3 (green). Nuclei, DAPI (Blue). Scale bar, 20 µm. The experiment was performed to similar results once. **b**, Cellular viral N gene RNA copy numbers in organoids treated with α-ACE2, mIFITM2 antibody blocking peptide and α-IFITM1-3 and infected with SARS-CoV-2 (MOI 0.15). Bars represent n=1. Related to Fig. 6.

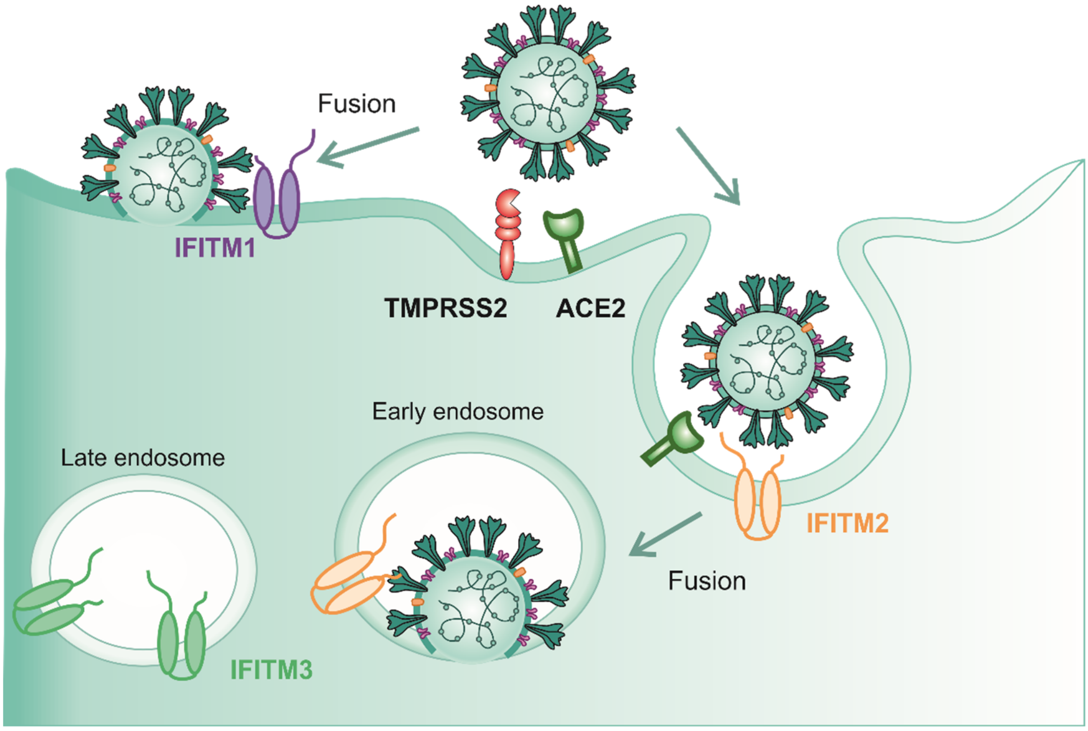

Supplementary Figure 13. Schematic presentation of the potential role of IFITM proteins in SARS-CoV-2 infection. SARS-CoV-2 particles use ACE2 as entry receptor ACE2. IFITM1 may facilitate attachment and fusion mainly at the plasma membrane, whereas IFITM2 may facilitate fusion predominantly in early endosomes. IFITM3 is localized to late endosomes. TMPRSS2, Transmembrane protease, serine 2. ACE2, Angiotensin-converting enzyme 2.

Supplementary Table 1. Oligonucleotides used in the study.

Name	Sequence and Dyes (5’-3’)
SARS-CoV-2 N Forward primer (HKU-NF)	TAA TCA GAC AAG GAA CTG ATT
SARS-CoV-2 N Reverse primer (HKU-NR)	CGA AGG TGT GAC TTC CAT G
Forward primer IFITM1	GGG CAT CCT CAT GAC CAT TGG A
Reverse primer IFITM1	GGC TAC TAG TAA CCC CGT TTT TCC TG
Forward primer IFITM2	GTC ACC ATG AAC CAC ATT GTG CAA AC
Reverse primer IFITM2	CCC CCA GCA TAG CCA CTT CC
Forward primer IFITM3	ACC ATG AAT CAC ACT GTC CAA ACC TT
Reverse primer IFITM3	CCA GCA CAG CCA CCT CG
SARS-CoV-2 N Probe (HKU-NP)	FAM-GCA AAT TGT GCA ATT TGC GG-TAMRA
Probe IFITM1	FAM/ZEN-ATC CTG TTA CTG GTA TTC GGC TCT GTG ACA GTC T-IBFQ
Probe IFITM2	FAM/ZEN-CTC CTG TCA ACA GCG GCC AGC CT-IBFQ
Probe IFITM3	FAM/ZEN-CTC TCC TGT CAA CAG TGG CCA GCC CC-IBFQ
