# Supplementary material for: IFITM proteins promote SARS-CoV-2 infection and are targets for virus inhibition in vitro
Source: Nat Commun. 2021 Jul 28;12:4584. doi: 10.1038/s41467-021-24817-y (PMC8319209; doi:10.1038/s41467-021-24817-y)
Supplement: Supplementary file 3 — Description of Additional Supplementary Files [file 41467_2021_24817_MOESM3_ESM.pdf]

Description of Additional Supplementary Files

File Name: Supplementary Data 1
Description: **Statistical calculations and exact p values**. Related to Fig. 1-6 and Supplementary Fig. 1-12.

File Name: Supplementary Movie 1
Description: **Localization of IFITM2/Spike PLA in early endosomes after 2h on ice and 15 min at 37°C.** 3D Reconstruction of confocal images of SARS-CoV-2 infected Calu-3 cells. Calu-3 cells stained with EEA1 (green), PLA (red) and DAPI-stained nuclei (blue). Colocalization of EEA1 and the Spike/IFITM2 PLA signal is highlighted in yellow. Related to Fig. 4.

File Name: Supplementary Movie 2
Description: **Localization of IFITM2/Spike PLA in early endosomes after 2h on ice.** 3D Reconstruction of confocal images of SARS-CoV-2 infected Calu-3 cells incubated 2h on ice. Calu-3 cells stained with EEA1 (green), PLA (red) and DAPI-stained nuclei (blue). Colocalization of EEA1 and the Spike/IFITM2 PLA signal is highlighted in yellow. Related to Fig. 4
