# Supplementary material for: IFITM proteins promote SARS-CoV-2 infection and are targets for virus inhibition in vitro
Source: Nat Commun. 2021 Jul 28;12:4584. doi: 10.1038/s41467-021-24817-y (PMC8319209; doi:10.1038/s41467-021-24817-y)
Supplement: Supplementary file 7 — Reporting Summary [file 41467_2021_24817_MOESM7_ESM.pdf]

nature research

Corresponding author(s): Frank Kirchhoff
Konstantin M.J. Sparrer

Last updated by author(s): 23.06.2021

Reporting Summary

Nature Research wishes to improve the reproducibility of the work that we publish. This form provides structure for consistency and transparency in reporting. For further information on Nature Research policies, see our Editorial Policies and the Editorial Policy Checklist.

Statistics

For all statistical analyses, confirm that the following items are present in the figure legend, table legend, main text, or Methods section.

n/a	Confirmed	
☐	☒	The exact sample size (*n*) for each experimental group/condition, given as a discrete number and unit of measurement
☐	☒	A statement on whether measurements were taken from distinct samples or whether the same sample was measured repeatedly
☐	☒	The statistical test(s) used AND whether they are one- or two-sided<br>*Only common tests should be described solely by name; describe more complex techniques in the Methods section.*
☒	☐	A description of all covariates tested
☒	☐	A description of any assumptions or corrections, such as tests of normality and adjustment for multiple comparisons
☐	☒	A full description of the statistical parameters including central tendency (e.g. means) or other basic estimates (e.g. regression coefficient) AND variation (e.g. standard deviation) or associated estimates of uncertainty (e.g. confidence intervals)
☐	☒	For null hypothesis testing, the test statistic (e.g. F, t, r) with confidence intervals, effect sizes, degrees of freedom and P value noted<br>*Give P values as exact values whenever suitable.*
☒	☐	For Bayesian analysis, information on the choice of priors and Markov chain Monte Carlo settings
☒	☐	For hierarchical and complex designs, identification of the appropriate level for tests and full reporting of outcomes
☒	☐	Estimates of effect sizes (e.g. Cohen's d, Pearson's r), indicating how they were calculated

Our web collection on statistics for biologists contains articles on many of the points above.

Software and code

Policy information about availability of computer code

Data collection	BD FACSDiva™ Version 8.0, ZEN 2010, Odyssey Licor
Data analysis	GraphPad Prism Version 8, Corel DRAW 19.1, LI-COR Image Studio Lite Version 3.1, ZEN 2010, Fiji

For manuscripts utilizing custom algorithms or software that are central to the research but not yet described in published literature, software must be made available to editors and reviewers. We strongly encourage code deposition in a community repository (e.g. GitHub). See the Nature Research guidelines for submitting code & software for further information.

Data

Policy information about availability of data

All manuscripts must include a data availability statement. This statement should provide the following information, where applicable:

- Accession codes, unique identifiers, or web links for publicly available datasets
- A list of figures that have associated raw data
- A description of any restrictions on data availability

A data availability statement is included, Raw data for Western blots is provided, Source data is included.

Field-specific reporting

Life sciences study design

All studies must disclose on these points even when the disclosure is negative.

Sample size	Sample sizes were chosen according to established protocols and not statistically assessed. Experiments were replicated, as indicated in the Statistics and Reproducability section or the corresponding figure legends usually at least three times independently to verify the results. For technical replicates usually only the mean is shown, unless otherwise indicated.
Data exclusions	no data was excluded
Replication	The number of independent replicates is indicated in the respective figure legends or the Statistics and Reproducability section to keep the main figure legends concise. All attempts at replication yielded similar results Experiments in Figure 1 were repeated independently three (a-c, e, g) to four times (d). Experiments in Figure 2a were performed twice. Experiments in Figure 2b-d were done three times. Experiments in Figure 3 a were repeated independently three times to similar results. Experiments in Figure 3 b-d were repeated independently two times to similar results. Experiments in Figure 4 a-d were repeated independently four times to similar results. Experiments in Figure 4 f-g were repeated independently two times to similar results. Experiments in Figure 5 were repeated independently three times to similar results. Experiments in Figure 6 a-b are representative experiments performed one time. Experiments in Figure 6 d were repeated independently two times to similar results. Replicates to similar results of the supplementary figures are indicated in the respective figure legends.
Randomization	Randomization was not applicable for this study, as no human trials or cohort studies were performed or samples assigned to experimental groups.
Blinding	Blinding was not applicable as results were quantified using machines and no human manual quantification was involved.

Reporting for specific materials, systems and methods

We require information from authors about some types of materials, experimental systems and methods used in many studies. Here, indicate whether each material, system or method listed is relevant to your study. If you are not sure if a list item applies to your research, read the appropriate section before selecting a response.

Materials & experimental systems

n/a	Involved in the study	
☐	☒	Antibodies
☐	☒	Eukaryotic cell lines
☒	☐	Palaeontology and archaeology
☒	☐	Animals and other organisms
☐	☒	Human research participants
☒	☐	Clinical data
☒	☐	Dual use research of concern

Methods

n/a	Involved in the study	
☒	☐	ChIP-seq
☐	☒	Flow cytometry
☒	☐	MRI-based neuroimaging

Antibodies

Antibodies used

α-ACE2 AK (AC18Z), Santa Cruz Biotechnology #sc-73668 (7.5µg/mL,15µg/mL,30µg/mL);
α-IFITM1 Cell Signaling #13126 S (1:1000), (7.5µg/mL,15µg/mL,30µg/mL)
α-IFITM2 Cell Signaling #13530S (1:1000),(7.5µg/mL,15µg/mL,30µg/mL)
α-IFITM3 Proteintech #11714-1-AP(7.5µg/mL, 15µg/mL, 30µg/mL),
α-IFITM1/2/3 (F-12) Santa Cruz Biotechnology #sc-374026 (7.5µg/mL, 15µg/mL, 30µg/mL),
α-IFITM3 Cell Signaling #59212S (1:1000)
α-SARS-CoV-2 N Sino Biologicals #40588-V08B (1:1000)
α-SARS-CoV-2 S (1A9) GeneTex #GTX632604 (1:1000)
α-IFITM2 Abcam #236735 (1:100)
α-VSV-M Absolute antibody #ABAAb01404-21.0 (1:1000)
α-Rab5 (RAB5A) Goat Polyclonal Antibody Origene #AB0009-200 (1:100)
α-RAB7 (RAB7A) Goat Polyclonal Antibody Origene #AB0033-200 (1:100)
α-EEA1 antibody - Early Endosome Marker abcam #ab206860 (1:100)
α-ACE2 Abcam, #ab166755 (1:1000)/(1:100)
α-V5 Cell Signaling #13202 (1:1000)
α-FLAG Sigma #F1804 (1:1000)
α-E-Cadherin Cell Signaling #24E10(1:200)
α-beta actin Abcam Cat#ab8226 (1:1000)
α-GAPDH Biolegend #607902 (1:5000)
α-Rabbit IgG (H+L) Secondary Antibody, HRP Thermo Fischer Cat#32460 (1:20000)
α-Mouse IgG (H+L) Secondary Antibody, HRP Thermo Fischer Cat#32430 (1:20000)
α-Rabbit IgG (H+L) Highly Cross-Adsorbed Secondary Antibody, Alexa Fluor Plus 488 Thermo Fischer Cat#A32731(1:20000)

	α-Mouse IgG (H+L) Highly Cross-Adsorbed Secondary Antibody, Alexa Fluor Plus 488 Thermo Fischer Cat#A32723(1:20000)<br>α-Mouse IgG (H+L) Highly Cross-Adsorbed Secondary Antibody, Alexa Fluor 647 Thermo Fischer Cat#A-31571(1:20000)<br>IRDye® 800CW Goat anti-Mouse IgG Secondary Antibody, Li-CORE, Cat#926-32210, lot C20808-02, dilution 1/10000<br>IRDye® 800CW Goat anti-Rat IgG Secondary Antibody, Li-CORE, Cat#926-32219, C91211-09, dilution 1/10000<br>IRDye® 680CW Goat anti-Rabbit IgG Secondary Antibody,Li-CORE, Cat#925-68071, lot C806605-11, dilution 1/10000<br>IRDye® 680CW Goat anti-Mouse IgG Secondary Antibody,Li-CORE, Cat#926-68070, lot C90910-21, dilution 1/10000<br>IRDye® 800CW Goat anti-Rabbit IgG Secondary Antibody,Li-CORE, Cat#926-32211, lot C70926-05, dilution 1/10000
Validation	Antibodies were validated by the manufacturer.

Eukaryotic cell lines

Policy information about cell lines

Cell line source(s)	Human HEK293T cells ATCC Cat# CRL-3216<br>Human epithelial colorectal adenocarcinoma cells (Caco 2)cells ATCC<br>Human epithelial lung adenocarcinoma cells (Calu-3 cells) ATCC<br>Mouse I1 Hybridoma CRL-2700; ATCC<br>Vero cells ATCC #CCL-81<br>Human human bronchial/tracheal epithelial (NHBE) Lonza<br>Human Small Airway epithelial cells (SAEC), Lonza<br>Human embryonic stem cell line HUES8 (Harvard University)<br>Human episomal hiPSCs Thermo fisher scientific #A18945<br>iPSC purchased from the Core facility of Cedars Sinai (Los Angeles, California)
Authentication	The cell lines were authenticated by ATCC, NIH or their lab of origin and not validated further in our laboratory.
Mycoplasma contamination	Cells were tested routinely to be free of mycoplasma using a PCR based test.
Commonly misidentified lines<br>(See ICLAC register)	N/A

Human research participants

Policy information about studies involving human research participants

Population characteristics	For the human stem cells used to generate neuronal cells:hiPSCs were obtained from 3 donors without known pathologies. Control 1 is a 45 years old female; Control Biocat is a 65 years old male, and Control 4 is a 49 years old man.
Recruitment	N.A.
Ethics oversight	For human stem cells used to generate gut organoids: Robert-Koch Institute: Approval according to the stem cell law 29.04.2020/AZ 3.04.02/0084. a more detailed description can be found in the Materials & Methods section of the manuscript.<br>For the human stem cells used to generate neuronal cells:hiPSC generation was performed in agreement with the ethical committee of the Ulm University (approval Nr.0148/2009 and 265/12) and in compliance with the guidelines of the Federal Government of Germany. The use of human material was approved by the Declaration of Helsinki concerning Ethical Principles for Medical Research Involving Human Subjects. All participants gave informed consent for the study.

Note that full information on the approval of the study protocol must also be provided in the manuscript.

Flow Cytometry

Plots

Confirm that:

- [x] The axis labels state the marker and fluorochrome used (e.g. CD4-FITC).
- [x] The axis scales are clearly visible. Include numbers along axes only for bottom left plot of group (a 'group' is an analysis of identical markers).
- [x] All plots are contour plots with outliers or pseudocolor plots.
- [x] A numerical value for number of cells or percentage (with statistics) is provided.

Methodology

Sample preparation	HEK293T cells were seeded in a 12 well plate format. 24h post transfection and post seeding, cells were harvested using a scraper. Afterwards cells were washed three times with PBS and fixed with 100µl of Reagent A (FIX & PERM Fixation and Permeabilization Kit, Nordic MUbio) for 30 minutes at room temperature, washed three time with PBS and stained with

primary antibody diluted 1:20 in PBS or in Reagent B for 1 h at 4°C. Cells were washed three times with PBS and stained with secondary antibody for 1 h at 4°C. After several washing steps with PBS, cells were resuspended in 100µl of PBS.

Instrument	FACS Calibur or FACS Canto; BD
Software	FACSDiva, BD
Cell population abundance	Single living cells (90%)
Gating strategy	The gating strategy outlines are provided in Supplementary Figure S7 and S10

☒ Tick this box to confirm that a figure exemplifying the gating strategy is provided in the Supplementary Information.
